# The Mutation of the Genes Related to Neurovirulence in HSV-2 Produces an Attenuated Phenotype in Mice

**DOI:** 10.3390/v12070770

**Published:** 2020-07-17

**Authors:** Lei Liu, Jishuai Cheng, Tangwei Mou, Ying Zhang, Xingli Xu, Jingjing Zhang, Xueqi Li, Xiao Feng, Xiangxiong Xu, Yun Liao, Shengtao Fan, Lichun Wang, Guorun Jiang, Qihan Li

**Affiliations:** 1 Institute of Medical Biology, Chinese Academy of Medicine Science & Peking Union Medical College, Yunnan Key Laboratory of Vaccine Research and Development on Severe Infectious Diseases, Kunming 650118, China; liulei@imbcams.com.cn (L.L.); handsome@imbcams.com.cn (J.C.); moutangwei@imbcams.com.cn (T.M.); zhangy@imbcams.com.cn (Y.Z.); xinglixu@imbcams.com.cn (X.X.); zhangjingjing940115@imbcams.com.cn (J.Z.); angelkiki@imbcams.com.cn (X.L.); fengxiao@imbcams.com.cn (X.F.); xiangxiongxu@mail.ynu.edu.cn (X.X.); liaoyun@imbcams.com.cn (Y.L.); fst@imbcams.com.cn (S.F.); wlc@imbcams.com.cn (L.W.); jgr@imbcams.com.cn (G.J.); 2 School of Life Science, Yunnan University, Kunming 650118, China

**Keywords:** *RL1-LAT-*HSV-2, *LAT-*HSV-2, infection, immune response

## Abstract

HSV-2 (Herpes simplex virus type 2) is a critical viral agent that mainly causes genital herpes and life-long latent infection in the dorsal root ganglia. Gene modification via CRISPR/Cas9 Clustered regularly interspaced short palindromic repeat sequences/CRISPR associated 9) was used here to construct HSV-2 mutant strains through the deletion of fragments of the *RL1* (Repeat Long element 1) and/or *LAT* (Latency-associated Transcript) genes. The HSV-2 mutant strains *LAT-*HSV-2 and *RL1-LAT-*HSV-2 present different biological properties. The proliferation of *RL1-LAT-*HSV-2 in nerve cells was decreased significantly, and the plaques induced by *RL1-LAT-*HSV-2 in Vero cells were smaller than those induced by *LAT-*HSV-2 mutant and wild-type strains. The observation of mice infected with these two mutants compared to mice infected with the wild-type strain indicated that the mutant *RL1-LAT-*HSV-2 has an attenuated phenotype with reduced pathogenicity during both acute and latent infections and induces a stronger specific immune response than the wild-type strain, whereas the attenuation effect was not found in mice infected with the *LAT-*HSV-2 mutant containing the *LAT* gene deletion. However, the simultaneous mutation of both the *RL1* and *LAT* genes did not completely restrict viral proliferation in nerve cells, indicating that multiple HSV genes are involved in viral replication in the neural system. This work suggests that the HSV-2 genes RL1 and/or LAT might be involved in the virulence mechanisms in mouse infections.

## 1. Introduction

Human herpes simplex virus type 2 (HSV-2) is a member of the *Herpesviridae* subfamily *Alphaherpesvirinae* [1], which causes genital herpes in the primary infection and rapidly penetrates the neurons distributed in these tissues, traveling along the axons to the dorsal root ganglion for a life-long latent infection [2,3]. HSV-2 can also cause recurrent diseases in response to stimuli, such as ultraviolet radiation, fever, trauma [3,4]. HSV-2 infections result in a number of severe outcomes, including increased mother-to-child transmission of neonatal herpes or herpes simplex encephalitis [5,6], and increased morbidity and mortality in neonates with encephalitis [7,8]. Epidemiologic studies have suggested that HSV-2 increases the probability of HIV-1 acquisition by 3-5-fold and seriously affects health [9,10,11] and quality of human life. Currently, there are many antiviral drugs in clinical practice that inhibit HSV-2 virus replication, such as acyclovir (ACV), which has been shown to reduce the occurrence of HSV-2 lesions by approximately 50% [12,13]. However, no obvious clinical effect on the suppression of the latent virus has been observed with these antiviral drugs, and they have certain side effects [12,13]. Although there are many live attenuated vaccines and subunit vaccines in clinical trials, the absence of effective HSV-2 prophylactic or therapeutic vaccines suggests that the mechanism of HSV-2 infection and immunity needs to be further explored.

In our previous study, a preliminary analysis of an HSV-1 mutant (also belonging to *Alphaherpesvirinae*) generated using the CRISPR/Cas 9 method was performed and showed a favorable attenuation phenotype and immune protection [14,15]. In this study, a deficient mutant strain of HSV-2 was constructed using the same technology, with the prospect of preferable attenuation and immune effects. HSV is neurotropic [16,17], and its coding genes *RL1* and latency-associated transcript (*LAT*) are both necessary for HSV-2 to invade sensory neurons [17,18]. Our research focused on the neurovirulence factors *RL1* and *LAT* [17,18,19]. The ICP34.5 protein encoded by the *RL1* gene has been shown to reverse the shutoff of protein synthesis by targeting eIF2α activated by PKR (double-stranded RNAto reverse the shutkinase R) for dephosphorylation of the IFN (interferon)signaling pathway and could play an important role in nerve cells infected with HSV [20,21]. Furthermore, as the virus remains latent in the central nervous system, the *LAT* gene is still expressed. In many reports, it has been listed as an important factor for the establishment of latency [2], and the presence of microRNAs in the intron or exon regions suggests that this gene is involved in the effective transcription of the α, β, and γ genes [2,22,23]. In our previous study, we constructed an HSV-2 mutant strain by partially deleting the *RL1* gene and found that *RL1-*HSV-2 did not have significantly decreased proliferation and pathogenicity. Furthermore, the partial deletion caused more serious pathological effects and showed key transcriptional regulation functions of viral genes associated with proliferation. We hypothesized that HSV-2 infection of nerve cells could be greatly alleviated by the simultaneous deletion of these two neurotropic genes, thus reducing the virulence of HSV-2, as well as its acute and latent infection abilities. To verify this hypothesis, we constructed two mutant strains, *LAT-*HSV-2 and *RL1-LAT-*HSV-2, using CRISPR/Cas9 genetic recombination modification technology. We explored whether the dual ablation of *RL1* and *LAT* genes causes low proliferation characteristics in nerve cells and whether it causes pathological physiological function changes in infected animals and plays an important role in the T cell changes in infected mice. These results provide an in-depth exploration of the *RL1* and *LAT* genes and provide a new perspective for basic research on HSV-2 and the construction of attenuated strains.

## 2. Materials and Methods

### 2.1. Cells and Viruses

An African green monkey kidney cell line (Vero; ATCC, Rockefeller, MD, USA), a human neuroblastoma cell line (SH-SY5Y; ATCC, Manassas, VA, USA), a human embryonic kidney cell line (HEK293T; ATCC, Manassas, VA, USA), and a human glioma cell line (HS683; ATCC, Manassas, VA, USA) were cultivated in Dulbecco’s modified Eagle’s medium (DMEM; Corning, Shanghai, China) containing 10% fetal bovine serum (FBS; HyClone, Glendale, AZ, USA). The medium was transformed to DMEM containing 2% FBS after viral infection. The wild-type HSV-2 strain HG52 was purchased from Wuhan Virus Institute (Wuhan, China), and the mutant RL1-HSV-2, LAT-HSV-2 and RL1-LAT-HSV-2 strains were constructed by our experiments.

### 2.2. Mice and Ethics

Four-week-old female BALB/c (BALB/c is an albino, laboratory-bred strain of the House Mouse from which a number of common substrains are derived.) mice were purchased (Vital River Laboratory Animal Technology Company, Beijing, China) and housed in a specific pathogen-free facility at the IMB, CAMS (Institute of Medical Biology, Chinese Academy of Medicine Science). Animal experiments were designed based upon the principles expressed in the “Guide for the Care and Use of Laboratory Animals” and “Guidance for Experimental Animal Welfare and Ethical Treatment” [15]. All animals were under the full care of veterinarians at the IMB, CAMS. The animal experimental protocols were reviewed and approved by the Experimental Animal Ethics Committee of the Institute (approval number: DWSP201803018-1, 1 March 2018) [15].

### 2.3. Plasmid Construction and Transfection

gRNA plasmids were constructed on the basis of the protocol reported by Xu et al. [15]. gRNA dsDNA (gRNA-*LAT*-1, gRNA-*LAT*-2 and gRNA-*LAT*-3) fragments were formed a double chain and inserted into the Bbs1 (New England Biolabs, Ipswich, MA, USA) site of the PX330 (Addgene, Cambridge, MA, USA) vector including the CRISPR/Cas9 system. We used Fugene HD reagent (Promega, Madison, WI, USA) to transfect the plasmids into HEK293T cells according to the protocol. A *US4* gene fragment was ligated to a PGEM^®^-T vector (Promega, Madison, WI, USA) as the standard template for qPCR. The specific primers used are listed in Table S1.

### 2.4. Construction of Recombinant Mutant Viruses

According to the methods perfomed by Xu et al. [15], a recombinant *LAT-*HSV-2 virus with a *LAT* deletion mutation was constructed via cotransfection of the plasmids PX330-*LAT*-1 and PX330-*LAT*-2 using Fugene HD reagent (Promega, Madison, WI, USA) according to the protocol. A recombinant *RL1-LAT-*HSV-2 virus with a *LAT* deletion mutation was constructed via cotransfection of the plasmids PX330-*LAT*-2 and PX330-*LAT*-3 based on the *RL1-*HSV-2 constructed in our previous study, using Fugene HD reagent (Promega, Madison, WI, USA) according to the protocol. The virus was obtained from infected HEK293T cells (multiplicity of infection (MOI) = 1) at 48 h postinfection (p.i.), and viral genomic DNA was extracted from samples using an AxyPrep^TM^ Body Fluid Viral DNA/RNA Miniprep kit (Central Avenue Union City, CA, USA). The genomic region containing the CRISPR target site of the *LAT* gene was amplified by PCR using PrimeSTAR DNA polymerase (TaKaRa, Dalian, China) with the primers HSV-2-*LAT*-F and HSV-2-*LAT*-R. After the recombinant mutant band was determined by gel electrophoresis, the mutated viruses were purified through plaque assays.

### 2.5. Preliminary Analysis of the LAT-HSV-2, RL1-LAT-HSV-2 and HSV-2 Viruses

For the plaque assay, 10-fold serial viral dilutions ranging from 10^−2^ to 10^−6^ were prepared in DMEM without FBS. Diluted HSV-2, *LAT-*HSV-2 and *RL1-LAT-*HSV-2 viruses were cultivated in Vero cells covered with 2% methylcellulose (Solarbio, Beijing, China) in DMEM at 37 °C. At four days postinfection (d.p.i.), the cells were stained with 0.2% crystal violet (Beyotime, Shanghai, China) and the plaques were captured to assess their morphology. SH-SY5Y and HS683 cells were infected with *LAT-*HSV-2, HSV-2 or *RL1-LAT-*HSV-2 virus (MOI = 0.05) at 37 °C. The infected cells were collected at several time points (8, 16, 24, 32, 40, and 48 h p.i.), and the viral titers of all samples were detected using the standard 50% cell culture infectious dose (CCID_50_) virus titration method involving Vero cells [15].

### 2.6. Experimental Infection of Mice

In the acute infection period, BALB/c mice were infected via vaginal instillation or nasal instillation of 5 × 10^4^ plaque-forming units (PFUs) of *LAT-*HSV-2, *RL1-LAT-*HSV-2, HSV-2 or sterile phosphate-buffered saline (PBS, pH 7.4), which was used as a control. The mice were weighed every day, and the survival rate-to-mortality ratio was evaluated over a 16-d or 20-d period. Tissue samples of the mice were acquired immediately after death and assessed viral loads and organ pathology. To test the latent infection, BALB/c mice were infected via vaginal/nasal/footpad instillation of 5 × 10^3^ PFUs of *LAT-*HSV-2, *RL1-LAT-*HSV-2, and HSV-2. Tissue samples of the mice were acquired immediately after death and assessed viral loads and organ pathology at 1, 2 and 3 months.

### 2.7. Histopathology

Mouse organs were fixed in 10% formalin (Servicebio, Wuhan, China) and embedded in paraffin to obtain tissue blocks. Approximately two slides from each organ were stained with hematoxylin and eosin (H&E) for morphological analysis by light microscopy [15].

### 2.8. qRT-PCR

Mouse viral loads were detected by qPCR with absolute quantification. Based on the method performed by Kessler et al. [24], we selected the primers for the reaction from a highly conserved region of the gene *US4* in the HSV-2 genome as standard DNA samples. A standard curve was acquired using standard DNA samples. Viral genomic DNA was extracted from tissues using an AxyPrep^TM^ Body Fluid Viral DNA/RNA Miniprep kit (Central Avenue Union City, CA, USA). A probe (Sangon Biotech, Shanghai, China) was labeled with TAMRA at the 3′ end and 6FAM at the 5′ end. The reactions were performed by using Premix Ex TaqTM (Probe qPCR; TaKaRa, Dalian, China) on CFX96 Connect Real-Time System (Bio-Rad, Hercules, CA, USA). For the relative quantification of inflammatory cytokine expression in mice tissues, the expression levels were calculated by using the comparative Ct method (ΔΔ*C*t) with the mouse housekeeping gene GAPDH (glyceraldehyde-3-phosphate dehydrogenase). Gene expression is shown as the fold change (2^−ΔΔ*C*t^) relative to the levels in samples from the PBS-injected mice. The reactions were performed by using a One-Step SYBR Prime ScriptTM PLUS RT-PCR kit (TaKaRa, Dalian, China) [15]. The specific primers used are listed in Table S1.

### 2.9. Reactivation Analysis of Dorsal Root Ganglia In Vitro

Vero cells were added to a 12-well plate. After the cells had grown to form a monolayer, the original culture solution was removed and the cells were washed with PBS. Then, MEM (Minima Essential Medium) containing 10% FBS was added. Dorsal root ganglia were removed from the sacrificed mice 2 months post virus challenge. The tissues were cut into small sections and placed on monolayers of Vero cells. Cytopathic effects (CPEs) were monitored daily for 7 days [14].

### 2.10. Flow Cytometry Analysis and ELISPOT (Enzyme-Linked ImmunoSpot)

The spleen was isolated under sterile conditions, and the splenic lymphocytes were divided into lymphocyte suspensions according to the instructions of the lymphocyte separation solution (Dakewe Biotech, Beijing, China). For the flow cytometry analysis, the lymphocytes were stained with CD3 (BD, Franklin Lakes, NJ, USA) and detected by flow cytometry (LAR Fortessa, BD, USA). Flow JO software was used to analyze the total T cells populations of the lymphocytes. For the ELISPOT experiment, a mouse IFN-γ ELISPOT kit (MABTECH Inc., Cincinnati, OH, USA) was used according to the manufacturer’s protocol. Briefly, the plate was conditioned and seeded with splenic lymphocytes prior to the addition of the stimulant (95% pure peptides: gB498-505: SSIEFARL) (Sangon Biotech, Shanghai, China), which mainly target CD8^+^ T lymphocytes. The plates were then incubated at 37 °C for 12–48 h. After the incubation step, the cells and medium were removed, and the plate was developed. The colored spots were counted using an automated ELISPOT reader (CTL, Cleveland, OH, USA), with spot-forming cells (SFCs) representing HSV-2-specific IFN-γ-producing T cells [14].

### 2.11. Statistical Analysis

The data from the various assays, which were performed in triplicate, are expressed as the mean value with the standard deviation (SD). GraphPad Prism software 5.0 (San Diego, CA, USA) was used for the statistical analyses. The differences between each pair of groups were evaluated using SPSS 19.0. A survival analysis was performed to determine the survival rates of the infected mice [15].

## 3. Results

### 3.1. Modification and Preliminary Analysis of LAT-HSV-2 and RL1-LAT-HSV-2

**Figure 1:**
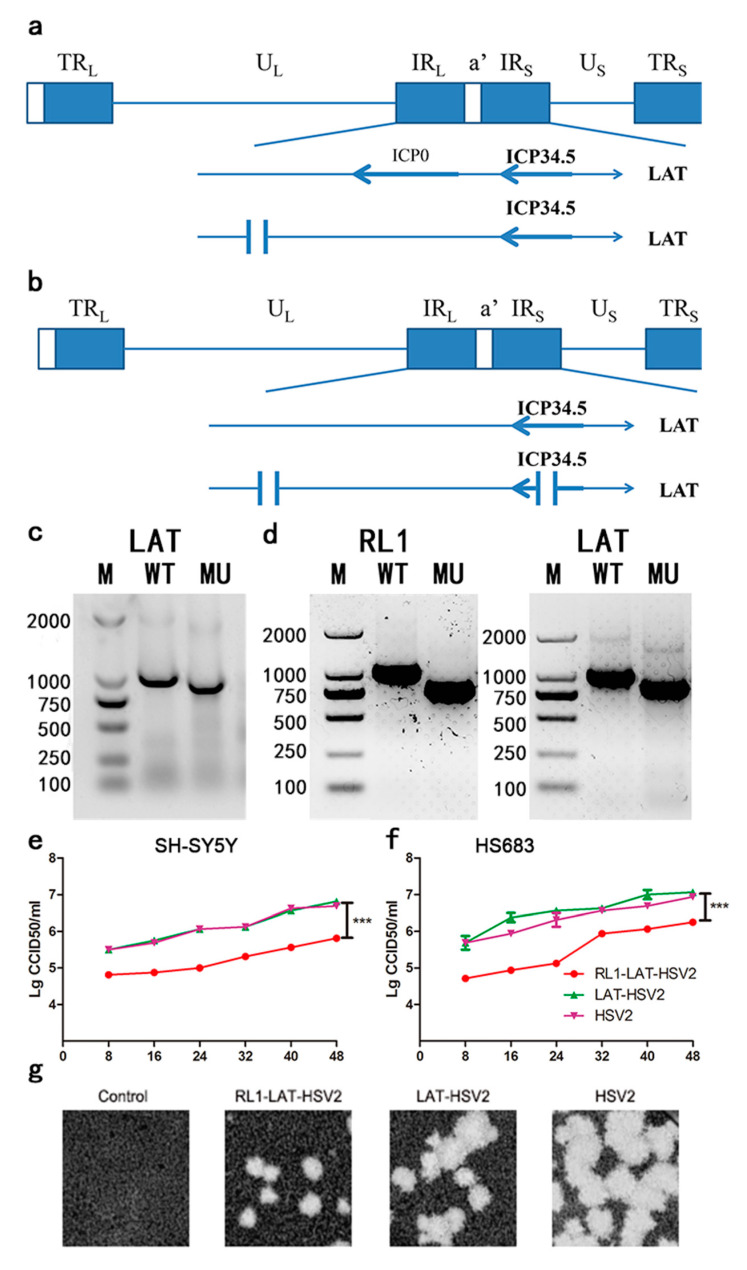
Modification of the *RL1* gene and *LAT* gene and identification of the *LAT-*HSV-2 and *RL1-LAT-*HSV-2 mutant strains. (**a**) The design and construction of the *LAT-*HSV-2 mutant strain, which contains a *LAT* gene with a 102 nt deletion generated by CRISPR/Cas 9 modification. (**b**) The design and construction of the *RL1-LAT-*HSV-2 mutant strain, which contains a *LAT* gene with a 190 nt deletion and an RL1 gene with a 240 nt deletion generated by CRISPR/Cas 9 modification. (**c**) The *LAT* gene was identified in the mutant (*LAT-*HSV-2) and wild-type (HSV-2) strains by PCR. (**d**) The *LAT* gene and *RL1* gene were identified in the mutant (*RL1-LAT-*HSV-2) and wild-type (HSV-2) strains by PCR. The proliferation of the mutant (*RL1-LAT-*HSV-2 and *LAT-*HSV-2) and wild-type (HSV-2) strains in human neuroblastoma (SH-SY5Y) cells. (**e**) The proliferation of the mutant (*RL1-LAT-*HSV-2 and *LAT-*HSV-2) and wild-type (HSV-2) strains in human neuroblastoma cell line (SH-SY5Y) cells. (**f**) The proliferation of the mutant (*RL1-LAT-*HSV-2 and *LAT-*HSV-2) and wild-type (HSV-2) strains in human glioma (HS683) cells. The data are shown as the mean ± SD. Statistical significance was measured by the log-rank test. *** *p* < 0.001. (**g**) Plaque phenotypes of the mutant (*RL1-LAT-*HSV-2 and *LAT-*HSV-2) and wild-type (HSV-2) strains in Vero cells stained using crystal violet. Magnification: 10×.

Various studies indicated that ICP34.5 gene is antisense to *LAT* and that the *LAT* gene encodes for miRNAs that can regulate ICP34.5 expression [23,25]. These two genes are essential for latent viral infection and spontaneous reactivation, and a deficiency of *RL1* and *LAT* genes can lead to reduced viral proliferation in nerve cells [2,15,26]. Therefore, the aim of this experiment was to design gRNAs located in the intron fragment of the *LAT* gene in the wild-type HSV-2 strain (Figure 1a); the missing fragment involving a 102 nt (nucleotide) deletion sequence of the *LAT* gene is shown in Figure 1c. The generation of *RL1-LAT-*HSV-2 involved the deletion of both the introns and exons of the *RL1* and *LAT* genes with 190 nt sequence deleted from the LAT gene and a 240 nt sequence deleted from the *RL1* gene. The designed gRNAs targeting *RL1* and *LAT* genes in the deletion mutant strains were constructed as shown in Figure 1b, and the characteristics of the two mutant strains are shown in Figure 1d, the sequencing results of *RL1* and *LAT* genes are in the supplementary files. Also, the mRNA transcription levels of eIF2α and PKR are elevated in *RL1-LAT-*HSV-2 infected VK2 cells at 6 h, 12 h, 18 h compared with *LAT-*HSV-2 and wild type virus (Figure S1). In the neuroblastoma cell line SH-SY5Y and the glioma cell line HS683, there was no significant difference between *LAT-*HSV-2 and the wild-type virus (Figure 1e,f). Interestingly, the mutant strains missing the *RL1* and *LAT* genes showed a lower growth rate than the *LAT-*HSV-2 and wild-type virus strains in the above two kinds of cells (Figure 1e,f). Furthermore, the observation of plaques on Vero cells also confirmed this point (Figure 1g).

### 3.2. Reduced Clinical Symptom Severity in RL1-LAT-HSV-2-Infected Mice

**Figure 2:**
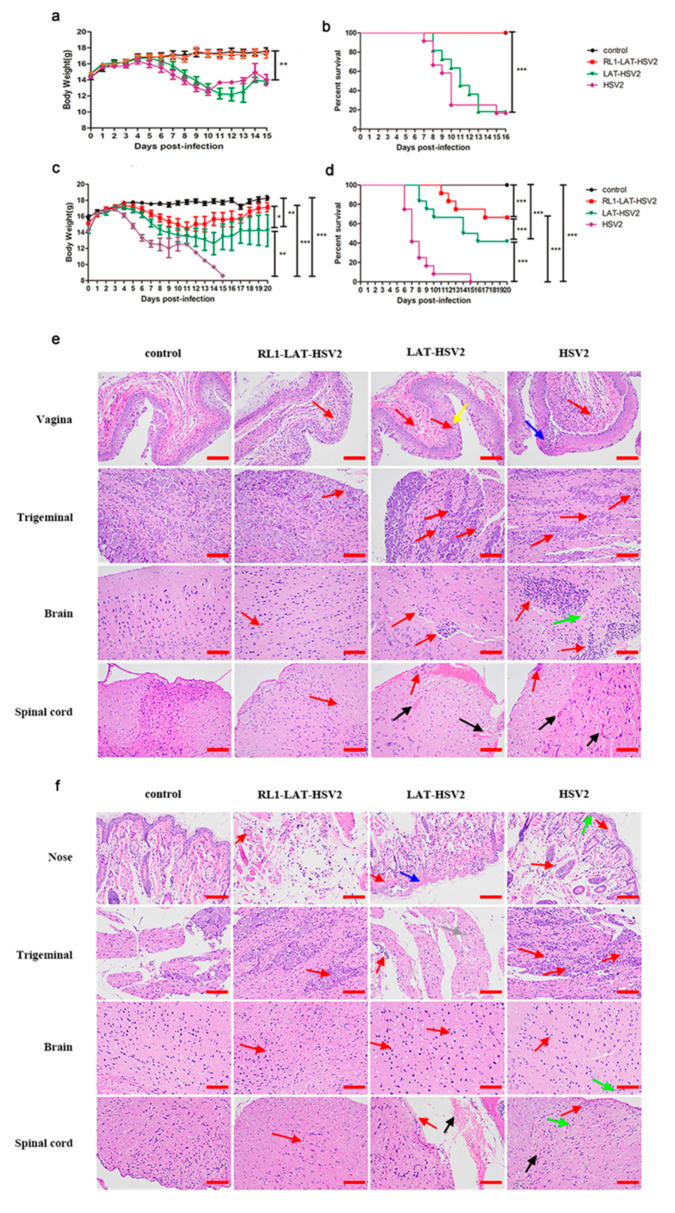
Clinical manifestations of mice infected with *RL1-LAT-*HSV-2 and *LAT-*HSV-2. (**a**) Body weight following vaginal challenge with *RL1-LAT-*HSV-2 (orange line), *LAT-*HSV-2 (green line) or HSV-2 (purple line). (**b**) Survival rate following vaginal challenge with *RL1-LAT-*HSV-2, *LAT-*HSV-2 or HSV-2. (**c**) Body weight following nasal challenge with *RL1-LAT-*HSV-2, *LAT-*HSV-2 or HSV-2. (**d**) Survival rate following nasal challenge with *RL1-LAT-*HSV-2, *LAT-*HSV-2 or HSV-2. (**e**) Pathological changes in the vaginal and nerve tissues of mice infected with *RL1-LAT-*HSV-2, *LAT-*HSV-2 or HSV-2 via vaginal challenge. Red arrows indicate the infiltration of inflammatory cells. Yellow arrows and blue arrows indicate the death of cells. Green arrows indicate the cytoplasmic vacuolization of cells. Black arrows indicate hyperemia. Samples were obtained at 5 days postinfection. Scale bar: 100 μm. (**f**) Pathological changes in the nose and nerve tissues of mice infected with *RL1-LAT-*HSV-2, *LAT-*HSV-2 or HSV-2 via nasal challenge. Red arrows indicate the infiltration of inflammatory cells. Blue arrows indicate the death of cells. Green arrows indicate the cytoplasmic vacuolization of cells (Cytoplasmic vacuolization can affect the normal function of cells, it is a symbol that cells are in poor condition and can even lead to cell death). Black arrows indicate hyperemia. Gray arrows indicate loose cytoplasm (Loose cytoplasm means cellular swelling and damage). Samples were obtained at 5 days postinfection. Scale bar: 100 μm. The data are shown as the mean ± SD. Statistical significance was measured by the log-rank test. * *p* < 0.05, ** *p* < 0.01; *** *p* < 0.001.

HSV mainly infects the individual through the genital mucosa and respiratory mucosa, causing severe herpes lesions after infection, and mouse models are extensively used due to their indirect simulation of HSV-2 infection. Previous studies have shown that paralysis of the hind limbs, redness and swelling of the vagina, or even death occurs in mice infected with the HSV-2 wild-type strain [1]. Here, we compared the three viruses *RL1-LAT-*HSV-2, *LAT-*HSV-2 and HSV-2 administered at a dose of 5 × 10^4^ PFUs via the nasal mucosa and genital tract, and clinical observations were made within 15 days of virus infection (Figure 2). When the mice were challenged via the genital tract, the body weight decline in *LAT-*HSV-2-infected mice was almost the same as that in HSV-2-infected mice, both approximately 20%, and the survival rates of the two groups were essentially the same, with survival rates of 10% within two weeks (Figure 2a,b). The body weight of the animals showed a continuous increasing trend in the *RL1-LAT-*HSV-2 group with a survival rate of 100%, similar to the control group (Figure 2a,b). However, when we infected mice with the same dose by nasal challenge, there were significant differences in the above clinical phenomena. After infection for four days, the *RL1-LAT-*HSV-2, *RL1-*HSV-2 and HSV-2 groups all showed significant weight loss. The body weight gradually recovered on the tenth day and fourteenth day in the *RL1-LAT-*HSV-2 group and *LAT-*HSV-2 group, respectively. However, the body weight of the *RL1-LAT-*HSV-2 group was always greater than that of the *LAT-*HSV-2 group (Figure 2c). In addition, after nasal challenge, although the *RL1-LAT-*HSV-2 group died (survival rate was 75%), the survival rate was significantly higher than that of the wild-type virus group (survival rate was 0) and *LAT-*HSV-2 group (survival rate was 50%; Figure 2d). Similarly, regardless of whether the mice were infected through vaginal or nasal routes, histopathological analyses showed less aggregation of inflammatory cells in the local tissues and nervous system (brain, spinal cord and sacral dorsal root ganglia) susceptible to HSV in the *RL1-LAT-*HSV-2 group than the wild-type group and *LAT-*HSV-2 group, which exhibited serious inflammatory cell aggregation, exudation, hyperemia and other pathological changes including cell death, cytoplasmic vacuolation, and loose cytoplasm, affecting the state and function of cells, causing cell damage and even death (Figure 2e,f). The pathological results of other tissues also matched the above conclusions (Table S2). In addition, on the first day after mice were infected by the vaginal route, the transcription level of inflammatory factors in the vaginal tissues of the *RL1-LAT-*HSV-2 group was lower than that of the LAT-HSV-2 group and the wild-type group (Figure S2).

### 3.3. RL1-LAT-HSV-2 Displays Reduced Viral Proliferation in Mouse Tissues

**Figure 3:**
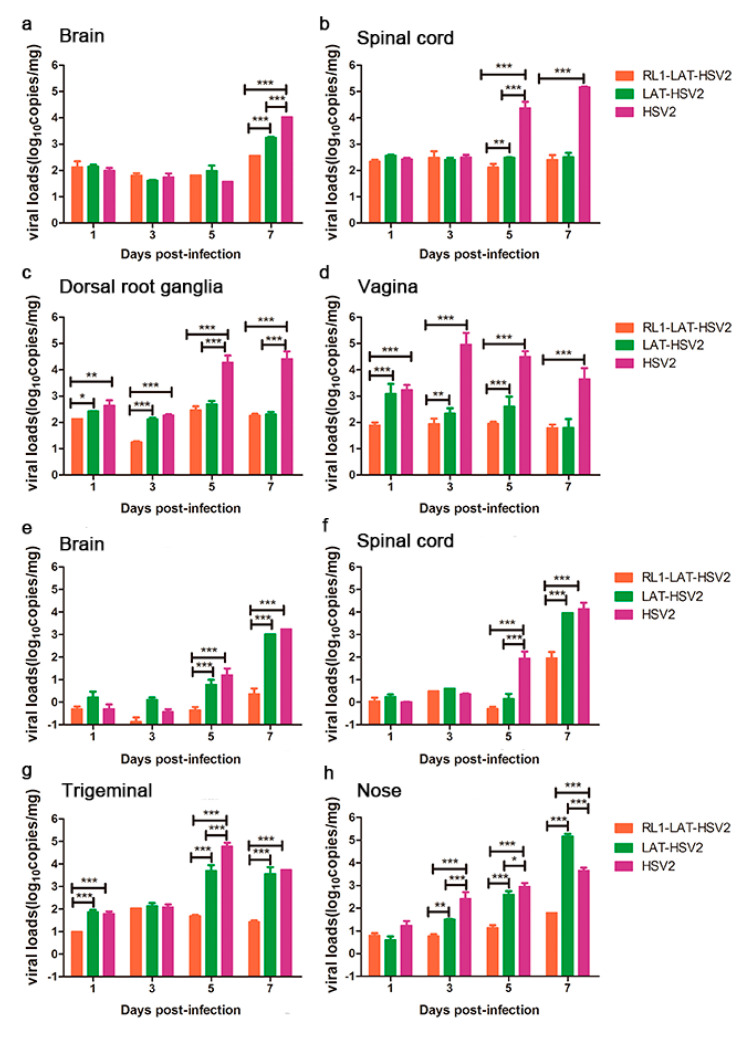
The proliferation of *RL1-LAT-*HSV-2 in various tissues from infected mice is reduced. Viral loads were determined in various tissues, including the brain (**a**), spinal cord (**b**), dorsal root ganglia (**c**), and vagina (**d**), from *RL1-LAT-*HSV-2, *LAT-*HSV-2 and HSV-2 vaginally infected mice. Viral loads were determined in various tissues, including the brain (**e**), spinal cord (**f**), trigeminal nerve (**g**), and nose (**h**), from *RL1-LAT-*HSV-2, *LAT-*HSV-2 and HSV-2 nasally infected mice. The data are shown as the mean ± SD. Statistical significance was measured by the log-rank test. * *p* < 0.05, ** *p* < 0.01; *** *p* < 0.001.

Previous data suggest that the pathological damage of nerve tissues and lesions induced by viruses are usually associated with the proliferation of the virus in animal tissues [14,15,27]. Thus, these tissues were collected and tested for their viral loads. The results showed that the *RL1-LAT-*HSV-2 group presented reduced viral proliferation consistent with the reduced severity of the clinical symptoms in both administration methods (Figure 3). After the vaginal challenge, there was no significant difference in the viral load in the brain tissue from days 1–5 in all three groups, but on the seventh day of virus infection when the mice began to die, the viral loads of the tissues in the *RL1-LAT-*HSV-2 group were two orders of magnitude lower than those in the wild-type virus group and one order of magnitude lower than those in the *LAT-*HSV-2 group (Figure 3a). The viral loads of the spinal cord and dorsal root ganglion tissues in the *RL1-LAT-*HSV-2 group were almost the same as those in the *LAT-*HSV-2 group. On the fifth and seventh days of infection, the viral loads of these two groups were two levels lower than those of the wild-type virus group (Figure 3b,c). The same trend was also observed in the mouse vaginal tissue at the challenge site, with the viral loads of the mouse tissues in the *RL1-LAT-*HSV-2 group being far lower than those in the *LAT-*HSV-2 group and the wild-type strain group (Figure 3d). After the nasal challenge, the viral loads of the *RL1-LAT-*HSV-2 group were significantly lower than those of the other two groups in the nerve tissue and the nasal tissue of the challenge site, which is similar to the results of the vaginal challenge (Figure 3e–h). In addition, the transcription levels of viral related genes in *RL1-LAT-*HSV-2 infected VK2 cells are lower than other two groups by at 6 h and 12 h (Figure S3).

### 3.4. The Latent Infection Capacity of RL1-LAT-HSV-2 Is Decreased

**Figure 4:**
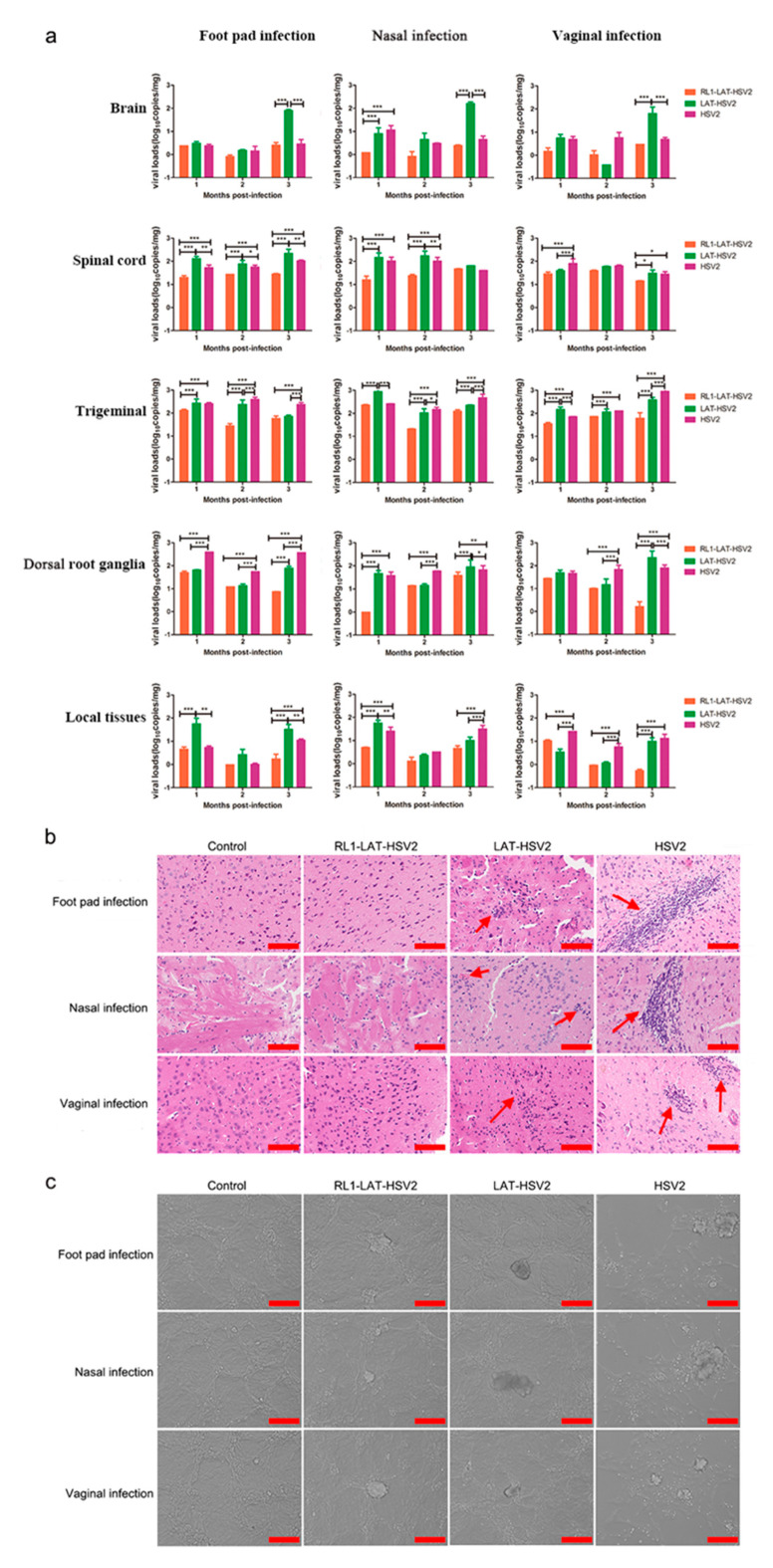
The latent infection capacity of *RL1-LAT-*HSV-2 is reduced in infected mice. (**a**) Viral loads were determined in various tissues, including the brain, spinal cord, trigeminal nerve, dorsal root ganglia and local tissues (footpad, nose and vagina), from *RL1-LAT-*HSV-2-, *LAT-*HSV-2 and HSV-2-infected mice subjected to footpad infection, nasal infection and vaginal infection. (**b**) Pathological changes in the brain tissue of mice infected with *RL1-LAT-*HSV-2, *LAT-*HSV-2 or HSV-2 via footpad challenge, nasal challenge and vaginal challenge. Red arrows indicate the infiltration of inflammatory cells. Samples were obtained at 2 months postinfection. Scale bar = 100 μm. (**c**) Vero cells cocultured with dorsal ganglia of infected mice at 2 months post viral challenge. Scale bars = 100 µm. The data are shown as the mean ± SD. Statistical significance was measured by the log-rank test. * *p* < 0.05, ** *p* < 0.01; *** *p* < 0.001.

The major feature of HSV-2 infection is that HSV-2 virus establishes latency in nerve ganglia [16] after viral infection; when infectious virus particles are cleared by the immune system, viral DNA in nerve cells cannot be completely cleared. However, it has been reported that the copy number of viral genomic DNA in the neural tissue of individuals with a latent infection is positively correlated with the severity of the latent infection [28]. Therefore, we administered mice a dose of 5 × 10^3^ PFUs *RL1-LAT-*HSV-2, *LAT-*HSV-2 and HSV-2 through the footpad, nose and vagina and then analyzed the pathological damage and viral proliferation in the nerve tissues and local tissues at 1 month, 2 months and 3 months, to produce a latency state similar to that of humans in a mice. The results showed that the postinfection proliferation in the tissues of the three challenge groups was not similar to that at 1–7 days after virus infection; furthermore, the unit copy number was less than 10^3^ copies/mg (Figure 4a). The viral load in all tissues and organs of the *RL1–LAT-*HSV-2 group was significantly lower than that of the wild-type virus and *LAT-*HSV-2 groups, especially in the trigeminal nerve, brain, spinal cord and virus infection site. However, the viral loads of the mouse tissues in the *LAT-*HSV-2 group were similar to those in the wild-type group, with no significant difference between the two groups. This was also proven by the pathological damage of the brain tissues in the three challenge groups (Figure 4b). After HSV-2 establishes latency in the dorsal root ganglia, when the body is subjected to nonspecific stimulation and reduced immunity, the latent virus in the nerve tissue is reactivated, and the symptoms of viral infection reappear. The difference between the viral infection of mice and humans is that spontaneous virus reactivation does not occur in mice infected with HSV; however, previous studies have shown that virus reactivation in mice can be detected by coculturing susceptible cells and trigeminal nerves infected with HSV-1 [29]. Therefore, we collected the dorsal root ganglia of mice challenged with either virus for 2 months and tested whether it could cause viral CPEs in Vero cells in vitro. The results showed that dorsal root ganglia of mice infected via any route with either *LAT-*HSV-2 or *RL1-LAT-*HSV-2 did not cause viral reactivation, whereas the dorsal root ganglia of mice infected with HSV-2 via any administration route were the did cause viral reactivation (Figure 4c and Figure S4). These results prove that removing both the *RL1* and *LAT* genes can reduce the latent viral infection ability; however, the phenomenon is not obvious when the *LAT* gene alone is lacking.

### 3.5. Increased Specific Immune Response Induced by RL1-LAT-HSV-2

**Figure 5:**
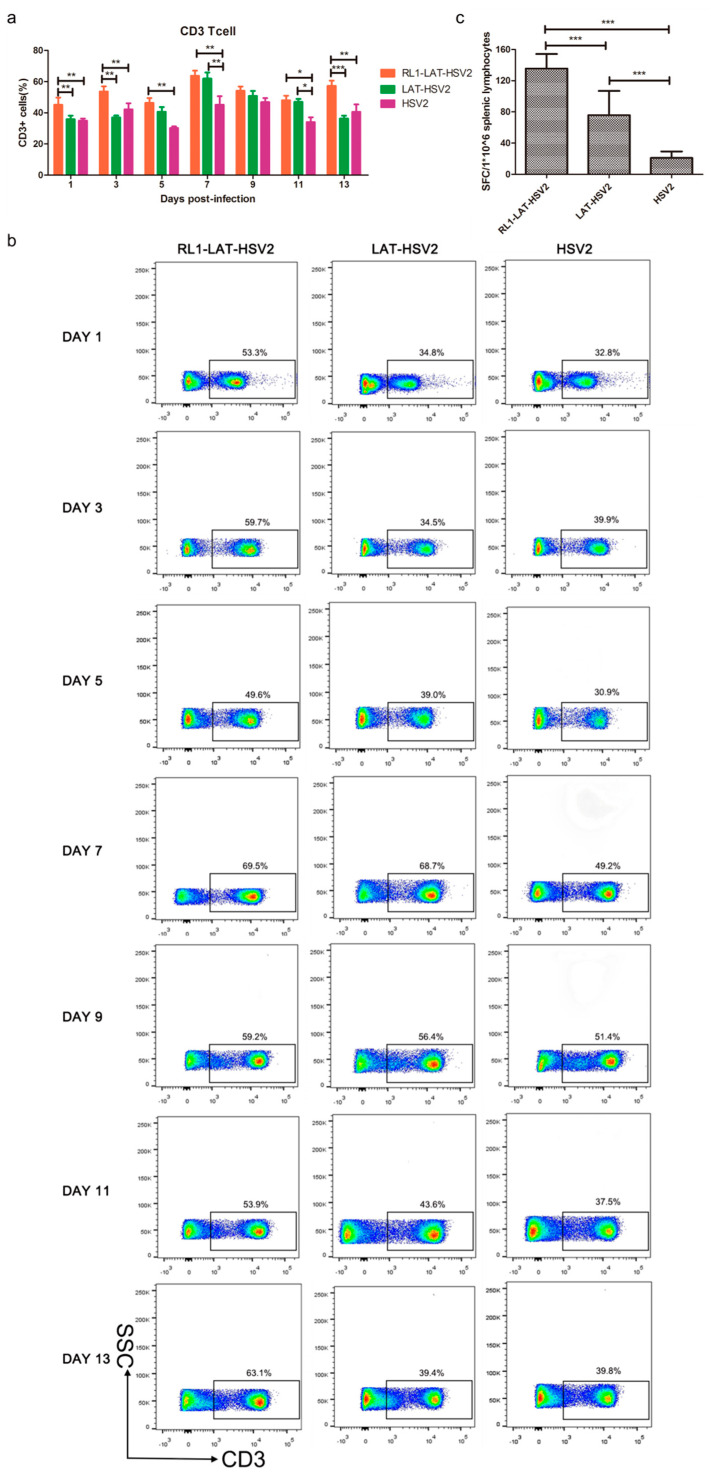
*RL1-LAT*-HSV-2 induces an increased specific immune response. (**a**) Statistics regarding the number of T cells among splenic lymphocyte cells in infected mice subjected to vaginal challenge. (**b**) T cells among splenic lymphocyte cells were detected by flow cytometry in infected mice subjected to vaginal challenge. The frame is the proportion of CD3^+^ T cells among splenic lymphocyte cells. (**c**) The ELISPOT responses showing IFN-γ-secreting cells among splenic lymphocytes from *RL1-LAT-*HSV-2, *LAT-*HSV-2 and HSV-2 vaginally infected mice at day 7 post viral challenge. The splenic lymphocytes were incubated for 24 h in the presence of the stimulus. The positive control was PHA (phytohemagglutinin). The data are shown as the mean ± SD. Statistical significance was measured by the log-rank test. * *p* < 0.05, ** *p* < 0.01; *** *p* < 0.001.

The findings of the above experiments are consistent with our initial hypothesis that deletion of the *RL1* and *LAT* genes can reduce the viral proliferation efficiency in nerve cells and acute and latent infection ability in the nerve tissues of animals; the decreased viral virulence is often caused by an enhanced immune response. Thus, we asked if the decreased *RL1-LAT-*HSV-2 viral loads in neural tissues are due to an increased immune response. We measured the variation in T lymphocyte numbers in mouse splenic lymphocytes within 1 to 13 days after viral infection. The results suggested that HSV-2 infection caused a decrease in the number of CD3^+^ T cells, and the number of CD3^+^ T cells in the splenic lymphocytes of mice infected with the RL1 and LAT mutant strains was significantly higher than that of mice infected with the wild-type virus or the *LAT-*HSV-2 virus (Figure 5a,b). To further explore the ability of *RL1-LAT-*HSV-2 infection to induce a mouse T cell-mediated immune response, we analyzed the number of viral antigen-specific T cells in peripheral blood mononuclear cells (PBMCs) in the spleens of mice by ELISPOT. The results indicated that on the 7th day after infection, the proportion of antigen-specific IFN-γ-producing T cells in the PBMCs of *RL1-LAT-*HSV-2-infected mice was greater than that in wild-type and *LAT-*HSV-2-infected mice (Figure 5c). These results suggest that *RL1-LAT-*HSV-2 is able to induce a strong specific immune response.

## 4. Discussion

HSV is double-stranded linear DNA virus with a large genome, and the amount of viral proteins encoded by HSV makes its structure and interaction with infected cells complicated [30,31,32,33]. Another characteristic of HSV is its ability to establish latency in nerve cells, which has hampered the development of HSV vaccines [2]. In previous studies, constructing mutant strains has been shown to be a vital method to explore the mechanism of the virus and its relevant vaccine patterns [34,35]. HSV encodes a variety of viral proteins that regulate viral infection and replication. Among them, the *RL1* gene encodes the ICP34.5 protein, named the “neurovirulence factor” in previous studies, which plays an important role in the toxicity of the virus to nerve cells [16]. The construction of an HSV-2 *RL1* deletion mutant by Katie L. Davis et al. demonstrated that RL1 deletion can reduce the proliferation capacity of the virus and enhance the IFN-mediated immune response [25]. The modification of the *LAT* gene, which is a latent transcription factor, can reduce the acute infection ability of the virus, and LAT gene deficiency in HSV-1 can also reduce the latent infection ability of HSV-1 [2,15]. We have successfully constructed *RL1-LAT-*HSV-2 mutant strain, and this mutant strain is absent of the capacity of inhibiting PKR and eIF2α in VK2 cells, indicating that the deletion of RL1 molecule can recover the host cellular stress-induced translational arrest. Our research hypothesis was that mutating HSV-2 *RL1* and *LAT* genes could decrease the infection ability of nerve cells. The experimental observations indicated that the mutant strain lacking both *LAT* and *RL1* genes exhibited significantly reduced proliferation in the HS683 glial tumor cell line and the SH-SY5Y neuroblastoma cell line, and the size of the plaques in Vero cells also proved that its virulence was reduced compared with that of the wild-type and *LAT-*HSV-2 strains. In BALB/c mice infected with *RL1-LAT-*HSV-2, during the acute infection period, the mutant strain group showed significantly less severe clinical symptoms than the two other groups. For example, the survival rate and weight decreased less in the *RL1-LAT-*HSV-2 group than the other two groups, and the virus caused only slight pathological damage. Furthermore, the local tissues and nerve tissues of mice infected with the mutant strains showed reduced proliferation, but the proliferation of *LAT-*HSV-2 was almost the same as that of the wild-type strain. However, when we administered BALB/c mice a low dose to simulate latent infection in humans, the DNA copy number, pathological damage and viral reactivation ability of the mutant strain were lower than those of the LAT-HSV-2 and wild-type virus strains, which also means that the HSV-2 mutant strain lacking both RL1 and LAT genes has a diminished ability to establish latency. Although the results of viral loads’ magnitude and pathology of *RL1-LAT-*HSV-2 are not the same in the different tissues of BALB/c mice through different challenge, under the same conditions, the overall trend of viral loads of *RL1-LAT-*HSV-2 groups is lower than that of the *LAT-*HSV-2 and HSV-2 groups, and the pathological damage is also weaker than that of the other two groups. The virus loads of *LAT* mutant strains in the brain and spinal cord tissues post latent infection even exceeds that of wild strains, this may be due to the strict suppression of virus lytic gene replication as the *LAT* gene is present [36,37], but when the *LAT* gene is deleted alone, the latent state may be broken, resulting in a small amount of lytic gene replication occurred in the virus, which increased the viral loads of *LAT-*HSV-2 infected mouse tissues. The results of the flow cytometry analysis and ELISPOT also demonstrated that *RL1-LAT-*HSV-2 induced an increased specific immune response, resulting in a reduced copy number of the virus. These results suggest that *RL1-LAT-*HSV-2 has an attenuated phenotype compared with that of the wild-type and *LAT-*HSV-2 strains and can cause an increased specific immune response. However, the attenuation effect is not significant when only the *LAT* gene is deleted. Previous data have indicated that RL1 assists successful HSV replication by regulating the host immune response [25], and the LAT gene is the only gene expressed largely during the HSV latent infection process [1]. RL1 can be targeted and regulated by *LAT* exon 2 miRNA (miR-1), which is vital for the induction of the latency period [23]; thus, *RL1* and *LAT* genes can affect viral establishment and maintenance of a latent infection in nerve cells. However, in this study, we found that when the neurovirulence of *RL1-LAT-*HSV-2 was decreased, the mutant strain pathogenicity and pathological damage were also significantly decreased, indicating that the *RL1* and *LAT* genes are associated with the nervous system and could also affect the replication and regulation of virus infection at different stages. This finding is in accordance with that of our previous work, which suggested that *RL1* is involved in the transcriptional regulation of the α and β genes. However, the fact that the simultaneous deletion of the *RL1* and *LAT* genes does not completely limit the proliferation of the virus in nerve cells indicates that many other genes participate in the replication and infection of HSV-2 in nerve cells. Furthermore, existing data indicate that it is feasible to explore the virus-attenuation mechanism by constructing HSV-2 mutant strains using *RL1* and *LAT* genes. This work may also provide a basis for HSV-2 vaccine research. On the basis of the decreased neurovirulence after knocking out the RL1 and *LAT* genes, we will later select *UL7* (genes related to virus proliferation) [15], *US3* and *US5* (genes ralated with cell apoptosis) [38,39] and *US12* (viral antigen presentation-related genes) [40] genes for deletion, which reduces the viral proliferation and enhances the host immune response against HSV-2, it is hoped that a candidate strain of live attenuated HSV-2 vaccine with both safety and protection will be developed in the future.

## Supplementary Materials

The following are available online at https://www.mdpi.com/1999-4915/12/7/770/s1. Figure S1. The mRNA levels of PKR and eIF2α in the VK2 cells infected with R*L1-LAT-*HSV-2, *LAT-*HSV-2 and HSV-2. Figure S2. The mRNA levels of various inflammatory factors in the vaginal tissue of mice infected with RL1-LAT-HSV-2, *LAT-*HSV-2 and HSV-2. Figure S3. The levels of viral genes in VK2 cells infected by RL1-LAT-HSV-2, *LAT-*HSV-2 or HSV-2. Figure S4. a. The levels of viral genes in co-culturing samples of Vero cells infected and dorsal root ganglia tissues from mice infected with *RL1-LAT-*HSV-2, *LAT-*HSV-2 or HSV-2. b. The viral titers of co-culturing samples of Vero cells and dorsal root ganglia tissues from mice infected with *RL1-LAT-*HSV-2, *LAT-*HSV-2 or HSV-2. The samples were obtained 7 day after co-culturing. The dorsal root ganglia tissues of mice were collected 2 month after infection. Table S1. Specific primers used in the experiment. Table S2. Pathological changes in other tissues of mice infected with *RL1-LAT-*HSV-2, *LAT-*HSV-2 and HSV-2.
