# Supplementary material for: The Mutation of the Genes Related to Neurovirulence in HSV-2 Produces an Attenuated Phenotype in Mice
Source: Viruses. 2020 Jul 17;12(7):770. doi: 10.3390/v12070770 (PMC7412103; doi:10.3390/v12070770)
Supplement: Supplementary file 1 [file viruses-12-00770-s001.pdf]

Supplementary Materials

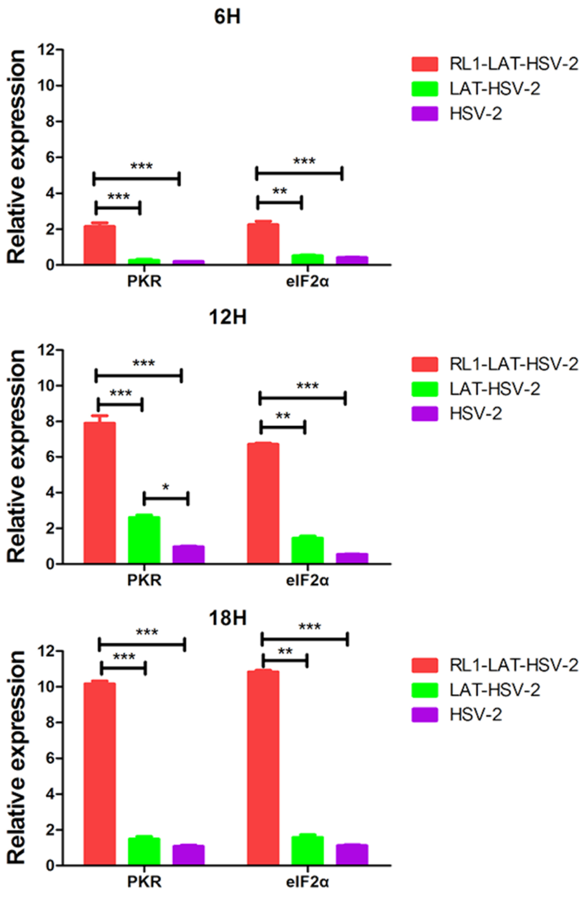

Figure S1. The mRNA levels of PKR and eIF2α in the VK2 cells infected with RL1-LAT-HSV-2, LAT-HSV-2 and HSV-2. The RNA levels of GAPDH were used to normalize the target RNA levels. Relative quantification was compared between the infected group and the control group (cells without infection).

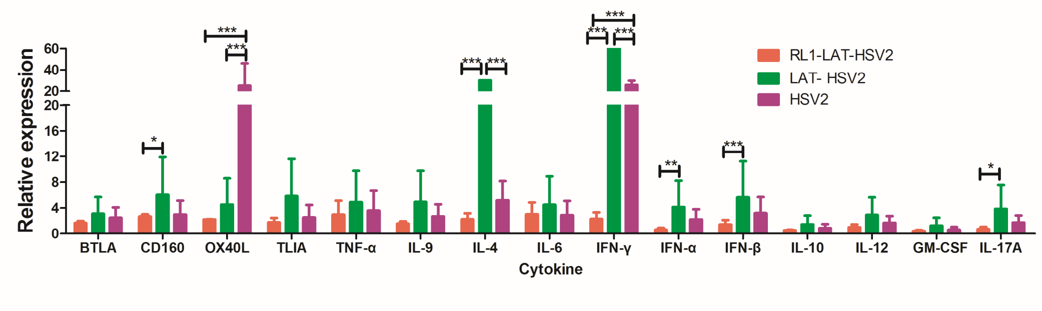

Figure S2. The mRNA levels of various inflammatory factors in the vaginal tissue of mice infected with RL1-LAT-HSV-2, LAT-HSV-2 and HSV-2. The RNA levels of GAPDH were used to normalize the target RNA levels. Relative quantification was compared between the infected group and the control

group (mice without infection). The samples were obtained 1 day after infection.

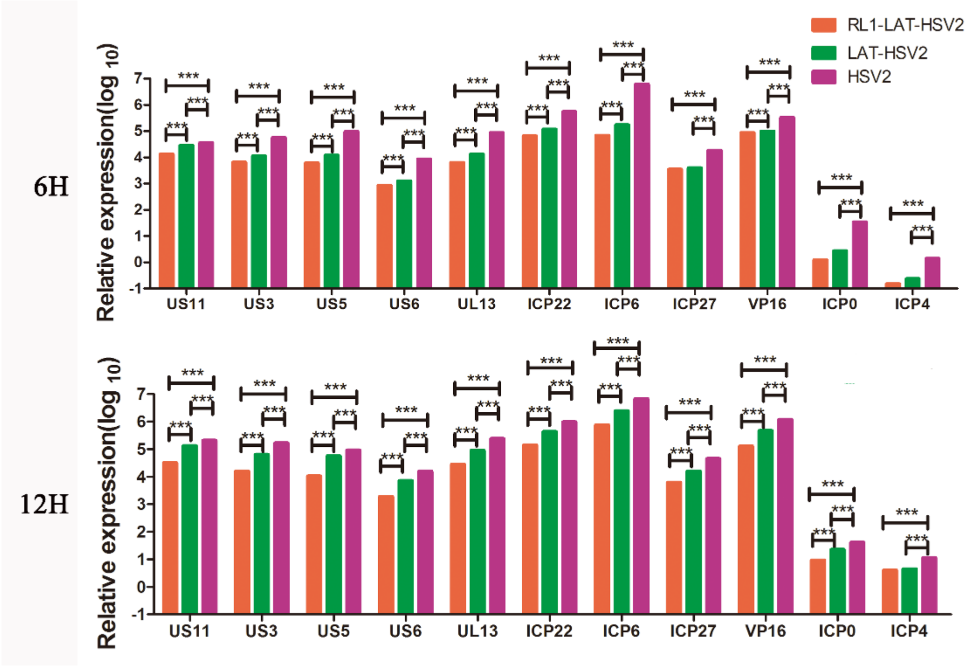

Figure S3. The levels of viral genes in VK2 cells infected by RL1-LAT-HSV-2 , LAT-HSV-2 or HSV-2.

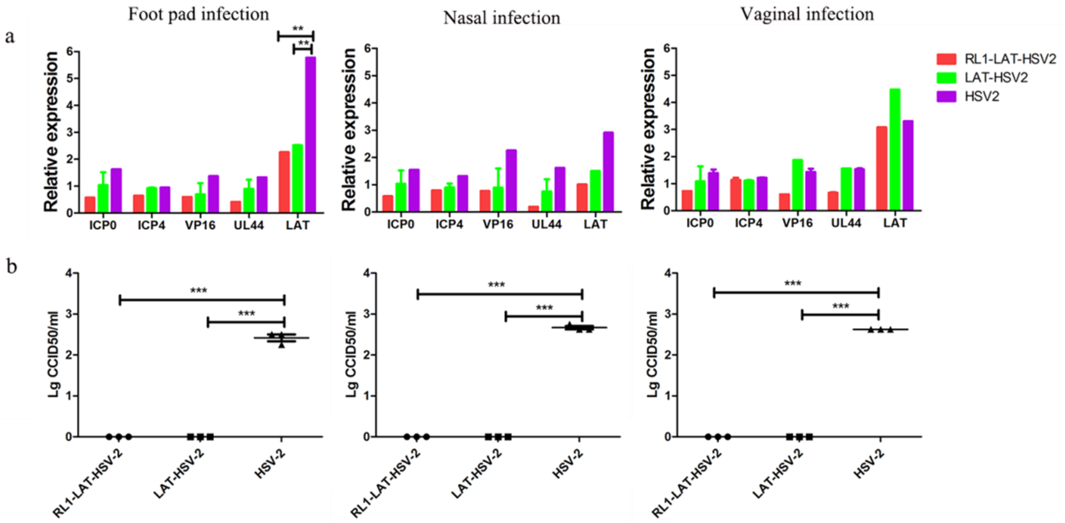

Figure S4. a. The levels of viral genes in co-culturing samples of Vero cells infected and dorsal root ganglia tissues from mice infected with RL1-LAT-HSV-2 , LAT-HSV-2 or HSV-2. b. The viral titers of co-culturing samples of Vero cells and dorsal root ganglia tissues from mice infected with RL1-LAT-HSV-2 , LAT-HSV-2 or HSV-2. The samples were obtained 7 day after co-culturing. The dorsal root ganglia tissues of mice were collected 2 month after infection.

Table S1. Specific primers used in the experiment.

Gene	Primer(5'-3')
gRNA-LAT-1-F	CACCGC GTTCTCGCAG GTAGGTTT
gRNA-LAT-1-R	AAACAAACCTACCTGCGAGAACGC
gRNA-LAT-2-F	CACCAGAG AGAAGAGAGC AGGGGT
gRNA-LAT-2-R	AAACACCCCTGCTCTCTTCTCTCT
gRNA-LAT-3-F	CACCAGCCGTC CGCCGGGGGA CCC
gRNA-LAT-3-R	AAAC GGGTCCCCCGGCGGACGGCT
HSV2-LAT-F	GAGAGGGACTCCGGAGAAGG AAGGC
HSV2-LAT-R	TGGATCCGAACACGCCGGATCCGCG
Mo-GAPDH-F	AGGTCGGTGTGAACGGATTTG
Mo-GAPDH-R	TGTAGACCATGTAGTTGAGGTCA
Mo-BTLA-F	GCCAGGACAGGAGAGTTA
Mo-BTLA-R	CTTACACCAAGTCACATTAGG
Mo-CD160 -F	CCTGAGACCAACTTAGAACA
Mo-CD160-R	AACACCAACTGAGATGACTT
Mo-OX40L F	TGCTTCTGTGCTTCATCTAT
M0-OX40L R	ATCTGGTAACTGCTCCTCT
Mo-TLIA F	AATAAGCAACAACTGGTTCC
Mo-TLIA R	ATTAGTCTGTCTCCTTCTTCC
Mo-TNF-α-F	GCCAACGGCATGGATCTCAA
Mo-TNF-α-R	TCTTGACGGCAGAGAGGAGG
Mo-IL-9-F	TGGTGACATACATCCTTGCCT
Mo-IL-9-R	GTGTCTCTGATGCCCCATGT
Mo-IL-4-F	GTGAGCTCGTCTGTAGGGCT
Mo-IL-4-R	CCGCTTACCGATGAATCCAGG
Mo-IL-6-F	TGGGACTGATGCTGGTGACA
Mo-IL6-R	ACAGGTCTGTTGGGAGTGGT
Mo-IFN-γ-F	ATCAGGCCATCAGCAACAACA
Mo-IFN-γ-R	CGTCTCACCTCAAACTTGGCA
Mo-IFN-α-F	TGGCGGTGCTGAGCTACTGG
Mo-IFN-α-R	TGTACCAGGAGTGTCAAGGCTCTC
Mo-IFN-β-F	GATGAACTCCACCAGCAGACAGTG
Mo-IFN-β-R	CACCATCCAGGCGTAGCTGTTG
Mo-IL-1β-F	TCGCAGCAGCACATCAACAAGAG
Mo-IL-1β-R	TGCTCATGTCCTCATCCTGGAAGG
Mo-IL-8-F	CCTGCTTGAATGGCTTGA
Mo-IL-8-R	GGCGGTATCTCTGTCTCT
Mo-IL-10-F	CTGCTATGCTGCCTGCTCTTACTG
Mo-IL-10-R	ATGTGGCTCTGGCCGACTGG
Mo-IL-12-F	CCTCCTGTGGGAGAAGCAGA
Mo-IL-12-R	CTTGAGCCTTTCAGGCGGAG
Mo-GM-CSF-F	AACATGTGTGCAGACCCGC

Mo-GM-CSF-R	TGGCTGTCATGTTCAAGGCG
Mo-IL-17A-F	GCAATGAAGACCCTGATAGA
Mo-IL-17A-R	TGCTGGATGAGAACAGAATT
Hu-PKR-F	TACCGTCAGAAGCAGGGAGT
Hu-PKR-R	GCAACCTACCTCCTATCATGTGG
Hu-eIF2α-F	ATGCCGGGTCTAAGTTGTAGATT
Hu-eIF2α-R	TCCAGCAAGCTGACATAAGCC
ICP0-F	CCTCCTCCGCCTCTTCCTCTG
ICP0-R	GCGTCTTCCTGGCACACTTCC
ICP4-F	ACCACCACCACCACCACCAC
ICP4-R	AGGAGGAGGCGGAGGAGGAG
VP16-F	CCAGCGTTCGGCAGCTACAC
VP16-R	GGTCTCGCGGTAGTACCTGTCC
UL44-F	ATGCCGGTTTCCCAACTCCA
UL44-R	CCTCTAAGCTAGGCGCCGTT
LAT-F	ACACGGGGCTGCCTTATACC
LAT-R	GGCGCATGCTAATGGGGTTC
US4-F	CGCTCTCGTAAATGCTTCCCT
US4-R	TCTACCCACAACAGACCCACG
US4-Probe	CGCGGAGACATTCGAGTACCAGATCG

Table S2. Pathological changes in other tissues of mice infected with RL1-LAT-HSV-2, LAT-HSV-2 and HSV-2.

Virus	Tissues	Day 1	Day 3	Day 5	Day 7
RL1-LAT-HSV2	Heart	++	+	+	+
	Liver	++	++	++	++
	spleen	+	+	+	+
	Lung	+	+	+	+
	Kidney	+	-	-	-
LAT-HSV2	Heart	++	++	+	+++
	Liver	++++	++++	++++	++++
	spleen	+++	+++	+++	+++
	Lung	++	++	++	++
	Kidney	++	+	+	-
HSV2	Heart	++	++	++	++
	Liver	+++	+++	+++	+++
	spleen	+++	+++	+++	+++
	Lung	++	++	++	++
	Kidney	+	++	++	++

—:Indicates no obvious abnormality

+:Indicates a small amount of inflammatory cells infiltration or bleeding

++:Indicates medium inflammatory cells infiltration or bleeding

+++:Indicates lots of inflammatory cells infiltration or bleeding

++++:Indicates serious inflammatory cells infiltration, bleeding or cell death
